# Behavioural consistency across metamorphosis in a neotropical poison frog

**DOI:** 10.1007/s10682-023-10274-0

**Published:** 2023-10-13

**Authors:** Lauriane Bégué, Noëlle Tschirren, Mélissa Peignier, Birgit Szabo, Eva Ringler

**Affiliations:** 1 Division of Behavioural Ecology, Institute of Ecology and Evolution, University of Bern, Bern 3032, Switzerland

**Keywords:** Anura, Personality, Exploration, Boldness, Development, Repeatability

## Abstract

Animals often show consistency in their behavioural repertoire across time and/or contexts that differs from other individuals of the same population, i.e. animal personality. We currently have quite an incomplete understanding of the factors that lead to behavioural traits remaining stable – or becoming decoupled – over an animal’s lifetime. In this study, we investigated the role of metamorphosis in the development of animal personality in a Neotropical poison frog, a species that undergoes drastic morphological and ecological changes during its development. We used lab-reared individuals of the brilliant-thighed poison frog *Allobates femoralis* to assess if consistent individual differences are already present at the tadpole stage, and if these differences are maintained throughout metamorphosis. We found evidence for two personality traits, exploration and boldness, already present in *A. femoralis* tadpoles. Despite the drastic changes in morphology, physiology, and habitat in the transition from tadpoles to metamorphs, personality traits persisted throughout metamorphosis, suggesting a physiological and/or genetic basis for the measured behavioural traits. We also found that exploration and boldness related behaviours were correlated with growth speed. Very bold and explorative individuals took fewer days until metamorphosis compared to very shy and non-explorative ones, which is in line with the concept of a Pace-of-Life Syndrome. These findings provide important insights into the proximate mechanisms that generate personality in species with complex life cycles.

## Introduction

Behavioural variation allows animals to adapt to changing environmental conditions. However, in most species individuals do not exhibit the full range of behaviours present in a population, but show behavioural consistency across time and/or contexts and behavioural variation between individuals of the same population (Pigliucci 2005; Dingemanse and Réale 2005). Such consistent individual differences have been termed ‘animal personality’ (Gosling 2001; Réale et al. 2007). Animal personality is typically measured along five major axes: aggressiveness, exploration, boldness, activity and sociability; although this list is not exhaustive and two or more traits might be linked forming a behavioural syndrome (e.g. positive correlation of aggression and exploration- related behaviours; Sih et al. 2004, 2012). Personality traits are latent variables that we cannot measure directly, but affect various behavioural responses that we can assess in a standardized way. For example, the personality trait aggressiveness will affect behaviours such as the latency to approach a conspecific competitor, the speed of approach, or the number of attacks. Previous research clearly demonstrated that personality matters for individual fitness, through its impact on reproductive success, dispersal, foraging, predator avoidance, and survival (Smith and Blumstein 2008; Cote et al. 2010; Réale and Dingemanse 2010; Schuett et al. 2010; Sih et al. 2012; Wolf and Weissing 2012).

Consistent individual differences in behaviour might be maintained through a balance between mutation and selection favouring genetic polymorphism, through frequency-dependent selection leading to the coexistence of different personality types, and/or through a link to life-history strategies (de Jong and Gavrilets 2000; Dingemanse and Réale 2005; Wolf et al. 2007; Biro and Stamps 2008; Dingemanse and Wolf 2010; Wolf and McNamara 2012; Carere and Maestripieri 2013). To link personality with life-history strategies and understand if and how personality changes to maximize fitness at any given moment in an individual’s lifetime, we need to better understand how personality changes during ontogeny (Stamps and Groothuis 2010; Groothuis and Trillmich 2011; Cabrera et al. 2021). We can expect personality to be consistent over an individual’s life if it has a strong genetic and physiological basis that is robust across life, and/or if the same personality traits are beneficial throughout life. However, if an animal experiences major changes in their physiology and morphology and/or their natural and social environment, different personality traits across life (decoupled personality) might become adaptive (Groothuis and Trillmich 2011; Wilson and Krause 2012a).

Animals with complex life cycles, such as amphibians (and anurans in particular), experience drastic changes in morphology, physiology, ecology and behaviour during and after metamorphosis (Wilbur 1980; Bishop et al. 2006). Therefore, they represent powerful models for studying behavioural changes across development on a broad scale (Wilson and Krause 2012a). Anuran larvae are aquatic, not able to reproduce, and are mainly grazers rather than active foragers; while after metamorphosis, individuals are typically terrestrial, after some further maturation period start to reproduce, and generally feed on life and moving prey (Wilson and Krause 2012b). Research has already demonstrated the existence of different personality traits in amphibian larvae as well as adults (e.g. Wilson and Krause 2012b; Carlson and Langkilde 2013; Kelleher et al. 2018; Chaloupka et al. 2022; Peignier et al. 2022a) but how personality changes within the same individuals across metamorphosis is still poorly understood (e.g. Wilson and Krause 2012b).

The few studies conducted on indirectly developing species that go through metamorphosis (amphibians and holometabolic insects) have, so far, not provided a clear picture on the role of metamorphosis on personality. Some studies found no consistency of behavioural traits across metamorphosis (e.g. Hedrick and Kortet 2012; Müller and Müller 2015; Monceau et al. 2017; Amat et al. 2018), while others show that some personality traits remain consistent over these dramatic morphological and physiological changes in an individual’s life (e.g. Brodin 2009; Hedrick and Kortet 2012; Wilson and Krause 2012b; Rodrigues et al. 2016; Amat et al. 2018). Due to the limited number of studies on this topic, we still have a very incomplete understanding of the role of metamorphosis in the development of animal personality.

Our aim was to investigate if there is consistency in exploration and boldness-related behaviours across ontogeny in a species that undergoes drastic morphological and ecological changes during its development. Neotropical poison frogs are great models to study behavioural variation across their development. They exhibit a high diversity in spatial and social behaviours (Summers and Tumulty 2014; Stynoski et al. 2015; Pašukonis et al. 2019). In contrast to other anuran species that remain in close association with aquatic environments throughout their lifetime, adult poison frogs are highly terrestrial and only commute to water bodies for tadpole deposition (Ringler et al. 2013, 2018). Thus, ecological niches of tadpoles (aquatic) and adults (terrestrial) differ considerably, which might favour decoupled personality traits across development.

We used lab-reared individuals of the brilliant-thighed poison frog *Allobates femoralis* to assess if consistent individual differences are already present at the tadpole stage, and if these personalities are maintained throughout metamorphosis. In a previous study, the existence of personality was demonstrated for adult individuals in a wild *A. femoralis* population (Peignier et al. 2022a). In the present study, we followed individual tadpoles over metamorphosis and repeatedly assessed their behaviours in two different experimental setups. We decided to focus on exploration- and boldness-related behaviours, as previous studies in other species have found these traits were already present at the tadpole stage (e.g. Brodin et al. 2013; Carlson and Langkilde 2013, 2014). If metamorphosis does not substantially impact on the mechanisms that shape behaviour in *A. femoralis*, we expected to find exploration and boldness-related behaviours to be correlated before and after metamorphosis within individuals. Alternatively, personality might actually be decoupled across ontogeny, especially when ecological niches differ dramatically between life stages, as has been found in a number of holometabolic insects (Amat et al. 2018).

## Materials and methods

### Study species

*Allobates femoralis* is a Neotropical poison frog (Dendrobatidae, sensu AmphibiaWeb 2022), naturally occurring in the tropical lowland forests of the Amazon basin and Guiana shield. During the breeding season, which coincides with the local rainy season, males are highly territorial and call to attract females and repel male competitors (Hödl et al. 2004; Ringler et al. 2011). When ready to mate, females visit male territories, where males and females engage in a prolonged courtship sequence (Stückler et al. 2019). Females deposit clutches of about 20 eggs in the leaf litter, which are externally fertilized by the male. After 15–20 days of larval development, males carry all hatched tadpoles on their backs to nearby water sources (Ringler et al. 2013). Once deposited in a pond, tadpoles hide under leaves to avoid predators and find food (Montanarin et al. 2011). Under natural conditions, tadpoles take approximately 40–50 days to reach metamorphosis (Ringler et al. 2012).

### Housing conditions

We conducted the experiments between November 2021 and March 2022 at the Ethological Research Station of the Division of Behavioural Ecology from the University of Bern (Switzerland) using individuals from a captive colony. Original stock for this population was sampled in and exported from French Guiana in 2018 and 2019 in compliance with all legal requirements from the responsible French authorities (DEAL: Arrêté n°R03-2018-04-26-001 & Arrêté n°R03-2019-03-15-003). During the breeding season, we precisely tracked clutch deposition and larval development in our entire colony. For the experiment, tadpoles were removed from their respective home terrarium five days after they reached Gosner stage 20 (Gosner 1960; Peignier et al. 2022b) to ensure all tadpoles were old enough to show sufficient mobility, and that all individuals were tested at similar developmental stages. We randomly sampled 40% tadpoles (2–7 tadpoles) from 11 clutches (from 8 parent pairs) to use as focal individuals (total N = 46) that we raised singly in transparent plastic tubes for the purpose of individual identification. We put all tubes into 60 × 40 × 20 cm plastic crates (food-safe), filled with water (50% reverse osmosis water and 50% stagnant water with oak leaves), and added a water filter. The tubes (3 cm in diameter) were covered at the bottom with a mesh to allow water flow while preventing tadpoles from escaping. This setup was chosen to resemble natural conditions in a pool, where multiple tadpoles from multiple clutches occur in groups, but at the same time allow individual identification of tadpoles for repeated testing. As individual tadpoles approached metamorphosis (all four legs clearly visible), we transferred them to bigger 5 × 12 × 6 cm plastic boxes (housing boxes). To allow water flow, the boxes were perforated at the bottom and a net was glued on one side to allow individuals to emerge after they completed metamorphosis. These housing boxes were placed in the same 60 × 40 × 20 cm crate filled with water. Water temperature was kept around 26 ± 1 °C and light was on from 8am to 8pm. Water was replaced once a week and tadpoles were fed with dry nettle leaves ad libitum.

Once individuals completed metamorphosis, we provided a layer of clay pebbles and autoclaved oak leaves at the bottom of the housing boxes. We transferred the housing boxes to another 60 × 40 × 20 cm crate without water, but instead a layer of clay pebbles at the bottom. The new crate was placed inside a second room in which light (light phase from 8am to 8pm), temperature (set cycle between 21 and 28 °C) and rain (twice a day for 5 min) were automatically set to mimic natural conditions as present in their natural habitat in French Guiana. Individuals were fed every two days with collembola and wingless fruit flies. To track growth, we took a picture on top of scale paper after every trial. From these pictures, we measured total body size of tadpoles (head to tip of tail) and snout-to-urostyle length (SUL) of metamorphs to the closest of 0.1 cm using the software ImageJ (Schneider et al. 2012).

### Experimental procedure

We tested exploration- and boldness-related behaviours using two distinct tests described below, which were performed consecutively on the same day with a one-hour resting period between the tests (hereafter referred to as ‘test session’). For each individual, test sessions were repeated four times during the tadpole stage and three times after metamorphosis. The order of the two tests was the same for all individuals within the same session but different across sessions. We performed the first test session the day after the tadpoles had been collected, and performed the following test sessions every 6 ± 1 days. Tadpoles were moved from tubes into larger housing boxes on average 12 (±1) days after the fourth test session. When individuals had successfully completed metamorphosis, which was approximately 3 ± 1 days later, we continued testing (i.e. fifth test session). The following test sessions were conducted every 6 (±1) days in the afternoon at a temperature of 25.6 ± 0.3 °C. We cleaned all equipment used between individuals to remove remaining odour cues of conspecifics from previous test sessions.

#### Open Field Test

**Fig. 1:**
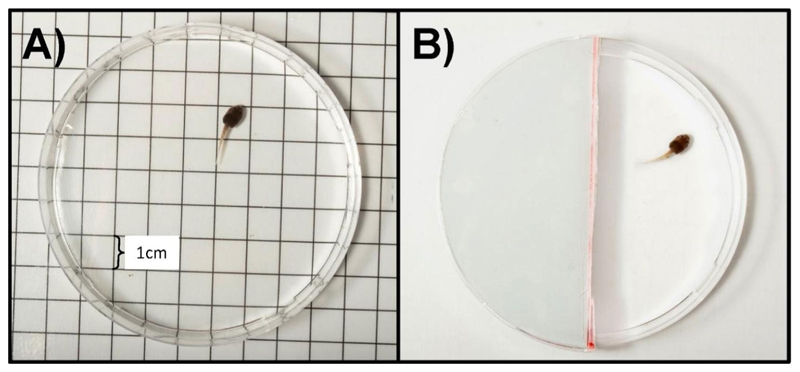
Experimental setups of the Open Field Test (**A**) and the Shelter Test (**B**)

We aimed to assess exploration-related behaviours of tadpoles and metamorphs using an Open Field Test. We put each individual in a Petri dish measuring 9 cm in diameter. For tadpoles, the Petri dishes were filled with 40 mL of water, while for metamorphs, the Petri dishes were empty. The sides of the dishes were taped with opaque paper to prevent visual distraction from outside movement. To prevent frogs from jumping out of the set-up, we elevated the sides to a height of 4 cm and closing the top with a transparent lid. The dishes were placed on top of a 1 × 1 cm grid (Fig. 1a) to facilitate tracking of all movements. Once an individual was placed inside the Petri dish, we covered it with a black piece of plastic for five minutes to allow them to acclimate. Then, we removed the cover and filmed the behaviour over a 10-minute period with a Sony Handycam HDR-PJ260 camera. We analysed the videos using the automated tracking software TOXTRAC (Rodriguez et al. 2018), to assess the distance travelled (in pixels). We assumed that this parameter was most relevant for assessing exploratory behaviour in tadpoles and metamorphs, for example in a foraging context. Distance travelled also was found to best represent exploration-related behaviours in a previous study in adult *A. femoralis* (Peignier et al. 2022a). For each individual we corrected the distance travelled by body size and used this corrected value in all further analyses.

#### Shelter Test

We aimed to assess exploration- and boldness-related behaviours of tadpoles and metamorphs using a Shelter Test (Fig. 1b) with similar settings as in the Open Field Test. In this test we placed individuals in a Petri dish and then covered the dish with an opaque lid that was cut in half for five minutes to allow tadpoles to acclimatise to the setup. Thereafter, we partially lifted both halves until we could determine on which side the individual was located. We then closed the half on the side the individual was located and removed the second half.

We recorded the individual’s behaviour for the subsequent 10 min using a Sony Handycam HDR-PJ260 camera. We used the coding software BORIS (Friard et al. 2016) to measure (i) the likelihood to exit the covered area (0 = not exited, 1 = exited), (ii) the latency (in s) to exit into the uncovered area, (iii) the time (in s) spent in the uncovered area and (iv) the number of crossings between covered and uncovered area. We gave a censored value of 600 s for the latency to exit into the uncovered area (occurrence in 28.9% of trials, 67 out of 232) and 0 s for the time spent in the uncovered area to individuals that did not exit the covered area during the entire test period. We expected that measures (i-iii) will reflect boldness-related behaviours, and variable (iv) exploration-related behaviours.

### Statistical analysis

We conducted all statistical analyses in R v3.6.0 (R Core Team 2020) using RStudio (RStudio Team 2020). To test if individuals show repeatable behaviours within and across life cycle stages, we used four data sets that either included data from tadpoles only (N = 184 tests from 46 individuals), metamorphs only (N = 51 tests from 17 individuals), individuals that we could successfully track from the tadpole to the metamorph stage (hereafter “tadpole-metamorph dataset”, N = 119 tests from 17 individuals), or all individuals regardless if they survived until metamorphosis or not (hereafter “full dataset”, N = 235 tests from 46 individuals). To conform to model assumptions (normality of residuals), we log transformed the latency to exit the covered area in the Shelter Test. We also used the function “transform-Tukey” from the package rcompanion (Mangiafico 2023) to apply a constant transformation on the time spent in the uncovered area in the Shelter Test, and on the distance travelled in the Open Field Test.

#### Repeatability

First, we investigated if *A. femoralis* tadpoles and metamorphs exhibit repeatability in behaviour. We assessed adjusted repeatability (R) of all measured behaviours in tadpoles and metamorphs separately using the “rpt” function in the rptR package (Stoffel et al. 2017). We fitted a separate model for each behaviour and included trial as a fixed effect and individual identity as a random effect in each. We estimated repeatability for the latency to leave the covered area and the time spent in the uncovered area in the Shelter Test, and the distance travelled in the Open Field Test from models fitted with a Gaussian error distribution. We estimated repeatability for the likelihood to exit the covered area in the Shelter Test from models fitted with a Binary error distribution, and for the number of crossings between covered and uncovered areas in the Shelter Test from models fitted with a Poisson error distribution. Behaviours were considered repeatable if the 95% confidence intervals did not overlap zero and models were run with 1,000 bootstraps.

#### Personality traits

Next, we determined how the behaviours are structured into different personality traits. Based on previous work in tadpoles (e.g. Brodin et al. 2013; Carlson and Langkilde 2013), we expected the best model to have two latent variables: one latent variable explaining the covariance between boldness-related behaviours (e.g., likelihood to exit the covered area, latency to exit the covered area and time spent in the uncovered area), and another latent variable explaining the covariance between exploration-related behaviours (e.g., number of crossings between covered and uncovered area, distance travelled in the Open Field Test). To test this, we used the full dataset and applied structural equation modelling using the SEM package on the phenotypic covariance matrix (Fox et al. 2020). Due to low sample sizes, we were not able to run the SEM for tadpoles and metamorphs separately. We scaled and centred all behavioural variables except for the likelihood to exit the covered area within stages to avoid issues with differences in behaviour based on testing in water (tadpoles) versus air (metamorphs). Furthermore, to avoid pseudo-replication, we derived the matrix from the means of each (non-transformed) behaviour across all trials for each individual. We built all possible models (e.g., with one or two latent variables, with or without interaction between the latent variables; electronic supplementary Table S1) and compared them based on their respective Akaike’s information criterion (AIC) to determine the most parsimonious model. Small values indicate higher parsimony and a ΔAIC ≥ 2 indicates a significant difference between models (Anderson and Burnham 2002).

#### Association with developmental time and consistency across trials and life stages –

To test if individual behaviour changes with age and/or due to habituation to the test setup, we used Bayesian generalised linear mixed models (GLMM, MCMCglmm package; Hadfield 2010) with the fixed effects ‘developmental stage’ (tadpoles or metamorphs), ‘trial’ (numeric, 1–4 for tadpoles and 1–3 for metamorphs), as well as their interaction. Additionally, we were interested if developmental time (days from Gosner stage 20 to metamorphosis; Gosner 1960) was associated with any of the behaviours measured in the tests by including the variable “days until metamorphosis” as another fixed effect. First, we built a binomial model using the likelihood to exit the covered area in the boldness test as the response variable. Then, we built three Gaussian models one with the latency to exit the covered area, one with the time spent in the uncovered area and one with the distance travelled in the Open Field Test as the response variable. Lastly, we built a Poisson model with the number of crossings between covered and uncovered areas as response variable. Each of these five models was performed on the tadpole-metamorph dataset including only the 17 individuals, which we could track throughout metamorphosis. For all models, we included individual and clutch identity as random effects. We investigated the results of the interactions using estimated marginal means of linear trends (emmeans package; Lenth 2023). For each MCMC model, we specified 2,000,000 iterations, a burnin of 100,000 and a thinning interval of 800. Furthermore, we used weak uninformative priors. We confirmed the absence of autocorrelation (checking that correlation between lags are < 0.1), sufficient mixing (via visual inspection of plots of MCMC chains), and that we ran the Markov chain for long enough (using the Heidelberg and Welch diagnostic tests; Hadfield 2010). We assumed statistical significance if the 95% credible intervals did not overlap 0. The absence of statistical significance in this analysis hence means that behaviours are not significantly different (i.e. behaviours are correlated) across developmental stages. To confirm our interpretation of the model results, we also conducted Spearman rank correlation tests of mean behaviour per developmental stage for all five behaviours.

## Results

### Repeatability

In tadpoles, the latency to exit the covered area, the number of crossings between covered and uncovered areas, and the time spent in the uncovered area were repeatable but had low scores that ranged from 0.15 to 0.21 (Table 1). In metamorphs, no behavioural variable was repeatable (Table 1). Due to low sample size and singularity issues, the repeatability of the likelihood to exit the covered area could not be estimated in metamorphs.

### Personality traits

**Fig. 2:**
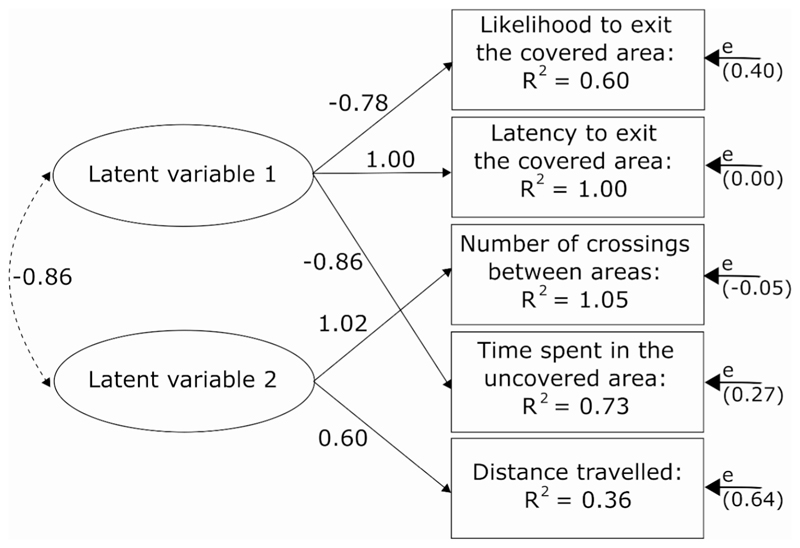
Path diagram of the best-structured equation model (SEM) (based on the difference in AIC values) explaining the covariance structure between the five behaviours assessed. R^2^ values represent the variances of the different behaviours explained by the SEM structure. Numbers associated with arrows represent how the behavioural response change based on changes to the latent variable (i.e., standardized factors loadings). Number in brackets represent error variances (e). The dashed arrow indicates a correlation between the two latent variables

Based on AIC, the best model supported the existence of a latent variable explaining the covariance between the likelihood and latency to exit the covered area, and the time spent in the uncovered area in the Shelter test. It also supported a second latent variable including the covariance of the number of crossings between covered and uncovered areas in the Shelter Test, and the distance travelled in the Open Field Test (model 16, AIC = 27.56; electronic supplementary Table S1). The two latent variables were negatively correlated (Fig. 2), however, the residual variances were high for some behaviours. The repeatability measurements and the results of the SEM together suggest that *A. femoralis* tadpoles and metamorphs exhibit one personality trait (which we define as ‘exploration’) encompassing the distance travelled, and the number of crossings between areas in a new environment. The results also suggest the existence of a second personality trait (which we define as ‘boldness’), encompassing the latency to leave a safe place and enter a new environment, the probability to enter a new environment and the time spent in an unknown environment.

### Association with developmental time and consistency across trials and life stages

**Fig. 3:**
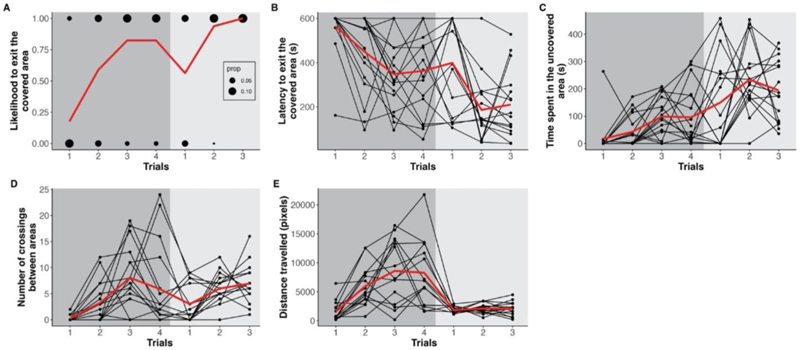
Change of behavioural responses per trial. (**A**) Likelihood to exit the covered area in the Shelter test. (**B**) Latency to exit the covered area in the Shelter test. (**C**) Time spent in the uncovered are in the Shelter test. (**D**) Number of crossings between areas in the shelter test. (**E**) Distance travelled in the Open Field test. Each dot represents one individual and the red line represents the median of all individuals. Only individuals who have been followed from the tadpole to the metamorph stage (N = 17) are shown here. Data for tadpoles are represented in trials 1–4 with a dark grey background and data from metamorphs in trials 1–3 with a light grey background

Individuals became more likely to exit the covered area in the Shelter Test across trials (GLMM, estimate = 5.362, CI_low_ = 1.893, CI_up_ = 9.435, p-value < 0.001; Table 2; Fig. 3a; electronic supplementary Table S2). Likewise, the latency to exit the covered area significantly decreased for both metamorphs and tadpoles across trials (GLMM, estimate = -0.359, CI_low_ = -0.578, CI_up_ = -0.149, p-value < 0.001) while the number of crossings between covered and uncovered areas (GLMM, estimate = 0.631, CI_low_ = 0.188, CI_up_ = 1.036, p-value = 0.003) and the time spent in the uncovered (GLMM, estimate = 3.229, CI_low_ = 0.436, CI_up_ = 6.216, p-value = 0.033) area increased (Table 2; Fig. 3b-d; electronic supplementary Table S2). Furthermore, tadpoles, but not metamorphs, increased the distance they travelled in the Open Field Test (Table 2; Fig. 3e; electronic supplementary Table S2). For all behaviours, values did not show significant differences across trials or developmental stages, except for the parameter ‘distance travelled’ (Table 2; Fig. 3; electronic supplementary Table S2). This was confirmed by correlation coefficients calculated from mean behaviour (Spearman rank correlation, R_exit_ = 0.51; R_latency_ = 0.42; R_crossings_ = 0.41; R_duration_ = 0.46; R_distance_ = 0.29). Finally, the days taken until metamorphosis were correlated with three behaviours. The latency to exit increased the more days tadpoles took until metamorphosis (GLMM, estimate = 0.024, CI_low_ = 0.006, CI_up_ = 0.043, p-value = 0.008). Contrary, tadpoles crossed between the light and dark area less often (GLMM, estimate = -0.042, CI_low_ = -0.084, CI_up_ = -0.001, p-value = 0.045) and spent less time in the uncovered area the more days they took until metamorphosis (GLMM, estimate = -0.299, CI_low_ = -0.498, CI_up_ = -0.089, p-value = 0.007; electronic supplementary Table S2).

## Discussion

Our results provide evidence for the existence of two personality traits, exploration and boldness, which are already present in *A. femoralis* tadpoles. Repeatability of behavioural traits was generally low, but variables remained stable across trials and developmental stages. Furthermore, we found a relationship between the latency to exit, number of crossings between areas and time spent in the uncovered area with the days taken until metamorphosis. These findings suggest that in *A. femoralis* personality is expressed already at the tadpole stage and persists throughout metamorphosis.

## Repeatability

In tadpoles, most of the behaviours that we assessed were repeatable and ranged from 0.15 to 0.21 (Table 1). These scores are lower than what has been found in most personality studies in other taxa (mean = 0.37, 95%CI = 0.35,0.38; Bell et al. 2009) or in a wild *A. femoralis* population (Peignier et al. 2022a). However, ectotherms (compared to endotherms) as well as captive individuals (compared to wild populations) are generally less repeatable in their behaviours (Bell et al. 2009) because of the strong effect temperature has on their behaviour. Even though we aimed to test individuals at consistent temperatures we cannot exclude unintended fluctuations in temperature affecting behaviour and increasing within-individual variation.

None of the measured behaviours were significantly repeatable in the metamorphs. When looking at the variation across trials in the metamorphs (trial 5–7), most parameters show high within-individual variation (Fig. 3a-d). However, given that the estimates fall in the same range as in the tadpoles (Table 1), we assume that the absence of repeatability in metamorphs is due to the relatively low sample size rather than a lack of personality. Only the parameter ‘distance travelled’ showed almost no variation, neither within nor between individuals. Theoretically, this could indicate some methodological issues that metamorphs were less likely to move in the Open Field Test setup. However, since metamorphs showed high mobility in the Shelter Test, e.g. in the variable ‘number of crossings between areas’, we do not assume that the test setup itself restricted the mobility of metamorphs.

The fact that lab-reared animals show generally less repeatability than individuals from wild populations could be due to low variation in environmental conditions during the early development, as breeding facilities typically follow a standardized procedure for animal husbandry. A study using tadpoles of the treefrog *Hyla intermedia*, demonstrated strong environmental effects on the development of tadpole personality; tadpoles that were reared under different biotic (predation risk) and abiotic (shading) conditions differed considerably in their behaviour at a later time point (Castellano and Friard 2021). Similarly, in agile frog (*Rana dalmatina*) tadpoles reared alone and without predator cues showed no evidence for repeatability or the existence of a behavioural syndrome, while the exposure to cues from predators and/or conspecifics resulted in the development of activity and risk-taking personalities and the existence of a behavioural syndrome (Urszán et al. 2015). Repeated social interactions with conspecifics (e.g. competition, help) during early development may also drive the differentiation of behaviour between individuals in a population (Beyts et al. 2023). All these results indicate that both the natural as well as the social environment during early life plays a key role in the development of behavioural profiles.

Future research should therefore aim to artificially diversify behavioural profiles of tadpoles. This could be achieved for example via experimental breeding lines (i.e. artificial selection), or via experimental manipulations of the rearing environment (high versus low disturbance/stress setup). Such longitudinal and experimental studies of personality will provide important insights into the proximate mechanisms that generate personality in species with complex life cycles.

### Personality traits

Using structural equation models, we showed that the five behaviours we measured were structured into two functional units (i.e. latent variables). The first latent variable included the covariance between the ‘likelihood and latency to exit the covered area’, and the ‘time spent in the uncovered area’ in the Shelter test, which we interpret as boldness-related behaviours. The second latent variable included the covariance of the ‘number of crossings between covered and uncovered areas’ in the Shelter Test, and the ‘distance travelled’ in the Open Field Test, which we interpret as exploration-related behaviours. The residual variances were high for some of the behaviours we measured (e.g. ‘likelihood to exit the covered area’, ‘distance travelled’, Fig. 2), which indicates that external factors that we did not measure also explain a part of the observed behavioural variation.

In the models, low factor scores for the latent variable ‘boldness’ indicate high levels of boldness (e.g. short latency to emerge from the covered area), while high factor scores for the latent variable ‘exploration’ indicate high levels of exploration (e.g. long distance travelled). Both latent variables were negatively correlated (Fig. 2), which suggests that highly explorative individuals were also very bold. Such a relationship between exploration and boldness has been found in many animal taxa in previous studies (Herde and Eccard 2013; Massen et al. 2013; Mazué et al. 2015), and hints towards shared underlying genetic and/or physiological mechanisms of these personality traits.

Interestingly, we also found that exploration and boldness related behaviours were correlated with growth speed. Very bold and explorative individuals took fewer days until metamorphosis compared to very shy and non-explorative ones. This finding is in line with the concept of a “Pace-of-Life Syndrome (POLS)”, which predicts a positive correlation between growth speed and several personality traits including boldness, activity, exploration, and aggression (Réale et al. 2010). Although the POLS was weakly supported by a recent meta-analysis (Royauté et al. 2018), differences found across taxonomic groups and sexes highlight that further empirical research is still urgently needed to better understand the link between morphological development and behaviour.

### Consistency across life stages

We did not find significant differences between behavioural variables across stages, which indicates that boldness- and exploration-related behaviours remain consistent within individuals, even across metamorphosis. Only the parameter ‘distance travelled’ showed significant differences between tadpoles and metamorphs, which could be due to the low between- and within-individual variation for this parameter at the metamorph stage (see trials 5–7 in Fig. 3e). Possibly, differences in foraging and anti-predator behaviour, or differences in the visual field of tadpoles and metamorphs (cf. Fouilloux et al. 2023) may have led to the observed differences in movement before and after metamorphosis. Unfortunately, very little is known about activity and movement patterns in juvenile poison frogs. Therefore, we are unable to tell whether the differences in exploration behaviour actually have any ecological relevance in our study species.

Research in various animal taxa has provided inconclusive results when looking at consistency of personality traits across different life stages (van Berkum et al. 1989; Monceau et al. 2017). The few previous studies in amphibians that have tried to track individual tadpoles throughout development, rarely found behavioural traits to be consistent across metamorphosis (Watkins 1997; Brodin et al. 2013; Castellano and Friard 2021). If animals are experiencing major re-organization of their body, including changes in their physiology, instability and/or decoupling of personality traits could be expected (Stamps 2007; Biro and Stamps 2008; Careau et al. 2008; Stamps and Groothuis 2010). Indeed, in species that undergo dramatic changes in their ontogenetic development, such as holometabolous insects, or amphibians during metamorphosis, consistency of individual behaviours across different life cycle stages are generally lower or totally absent (Müller and Müller 2015; Monceau et al. 2017; Amat et al. 2018; see also Wilson and Krause 2012a, b; Brodin et al. 2013).

Beside morphological re-organization, many animals require quite different habitats during their development, and might occupy contrasting ecological niches over their entire lifetime. On the one hand, highly contrasting environments during ontogeny might select for decoupled behavioural profiles in order to adapt to the current environmental conditions. On the other hand, similarities in habitat features or predation pressure across life stages might lead to selection favouring behavioural phenotypes to remain consistent (Réale et al. 2010; Biro and Stamps 2008; Cabrera et al. 2021). In the mealworm beetle *Tenebrio molitor*, which do not change habitat across metamorphosis, behavioural types were found to be decoupled across life stages (Monceau et al. 2017). Interestingly, in field crickets, sex differences in the repeatability of personality across metamorphosis was reported, which could be the result of differences in predation risk between males and females (Hedrick and Kortet 2012). In *A. femoralis*, habitat features drastically change across life stages and would support a decoupling of behavioural phenotypes, if being plastic would provide individuals with a strong selective advantage. However, we observed that behavioural traits remained consistent across tadpoles and metamorphs, which could indicate physiological/genetic constraints to plasticity and/or similar selection pressures acting on both life stages. For example, shy individuals might be more efficient in hiding from predators and thus benefit from increased survival in both, the tadpole and metamorph life stage. In turn, bolder or more explorative individuals might benefit from increased foraging and/or reproductive success (cf. Peignier et al. 2023). Future studies should investigate the heritability of personality traits in *A. femoralis*, to better understand the genetic basis for behavioural variation in this species.

## Conclusions

Individual *A. femoralis* undergo dramatic morphological and ecological changes over the course of their development. Still, we found behavioural traits to remain consistent across metamorphosis, which may suggest a strong physiological and/or genetic basis for the measured behavioural traits. Research in various animal taxa has provided inconclusive results about the role of metamorphosis or habitat differences across ontogeny in the development of animal personality. Future research should therefore aim to use controlled breeding designs together with experimental manipulations and genomic profiling to gain insights into the proximate mechanisms that generate personality in species with complex life cycles.

## Supplementary Material

Supplementary material

## Figures and Tables

**Table 1:** Repeatability (R) and confidence intervals (CI) of the behaviours measured. We calculated repeatability for each behavioural measure in the two tests separately based on data collected in tadpoles and metamorphs. Repeatability ranges from 0 to 1, with 0 indicating no repeatability (only within-individual variation) and 1 indicating very strong repeatability (only between-individual variation). Significant results are indicated in bold. NA – due to small sample sizes, R could not be estimated

Data	Behaviour	R	95%CI
**Tadpoles**	likelihood to exit the covered area	0.16	[0; 0.40]
	latency to exit the covered area	**0.15**	**[0.003; 0.30]**
	number of crossings between areas	**0.21**	**[0.01; 0.38]**
	time spent in the uncovered area	**0.19**	**[0.05; 0.35]**
	distance travelled	0.14	[0; 0.31]
**Metamorphs**	likelihood to exit the covered area	NA	NA
	latency to exit the covered area	0.17	[0; 0.5]
	number of crossings between areas	0.20	[0; 0.57]
	time spent in the uncovered area	0.01	[0; 0.34]
	distance travelled	0.27	[0; 0.60]

**Table 2:** Estimates and test statistics for the PostHoc comparison of the changes in behaviours across trials based on estimated marginal means of linear trends for tadpoles and metamorphs (tadpole-metamorph dataset). We assumed statistical significance if the confidence interval did not cross 0 which are highlighted in bold. CI – confidence interval

Response variable	Stage	Parameter	Lower 95%CI	Upper 95%CI
Likelihood to exit the covered area	metamorph	5.14	**1.89**	**9.43**
	tadpole	1.98	**1.09**	**3.13**
	*Difference between*			
	metamorph - tadpole	3.10	-0.18	7.19
Latency to exit the covered area (log transformation)	metamorph	-0.36	**-0.58**	**-0.15**
	tadpole	-0.21	**-0.34**	**-0.07**
	*Difference between*			
	metamorph - tadpole	-0.15	-0.41	0.10
Number of crossings between areas	metamorph	0.62	**0.19**	**1.04**
	tadpole	0.79	**0.48**	**1.11**
	*Difference between*			
	metamorph - tadpole	-0.15	-0.68	0.33
Time spent in the un-covered area (constant transformation)	metamorph	3.23	**0.44**	**6.22**
	tadpole	3.40	**1.60**	**5.19**
	*Difference between*			
	metamorph - tadpole	-0.18	-3.51	3.36
Distance travelled (constant transformation)	metamorph	-0.07	-1.92	1.74
	tadpole	2.26	**1.15**	**3.47**
	*Difference between*			
	metamorph - tadpole	**-2.32**	**-4.51**	**-0.20**

## Data Availability

All data generated during this study are available on the Open Science Framework (OSF; doi: https://doi.org/10.17605/OSF.IO/Q37HU).

## Code Availability

All code generated for analysis during this study are available on the Open Science Framework (OSF; doi: https://doi.org/10.17605/OSF.IO/Q37HU).
