# Supplementary material for: Behavioural consistency across metamorphosis in a neotropical poison frog
Source: Evol Ecol. Author manuscript; Available in PMC 2024 Jul 10. (PMC7616151; doi:10.1007/s10682-023-10274-0)
Supplement: Supplementary material [file EMS196914-supplement-Supplementary_material.pdf]

Supplementary material

to

"Behavioural consistency across metamorphosis in a neotropical poison frog"

by

Lauriane Bégué, Noëlle Tschirren, Melissa Peignier, Birgit Szabo, & Eva Ringler

Correspond to: Eva Ringler, Division of Behavioural Ecology, University of Bern, 3032 Bern, Switzerland; email: eva.ringler@unibe.ch,

Supplementary Tables

Table S1. List of all possible models built (e.g., with one or two latent variables, with or without interaction between the latent variables) and their respective Akaike's information criterion (AIC). a ΔAIC ≥ 2 was used to determine the most parsimonious model. We give details on model structure and AIC. Due to sample size issues not all models converged and some models were singular. Distance – distance travelled, out – excited the covered area, latency – time to exit, cross – number of crosses between areas, duration – time spent in the uncovered area.

Tadpoles only				
**Model number**	**Structure**		**AIC**	**Issue**
	**Regression**	**Covariance**		
0	-	distance <-> distance<br>out <-> out<br>latency <-> latency<br>cross <-> cross<br>duration <-> duration	181.69	none
1	Var -> distance<br>Var -> out<br>Var -> latency<br>Var -> cross<br>Var -> duration	distance <-> distance<br>out <-> out<br>latency <-> latency<br>cross <-> cross<br>duration <-> duration	31.52	none

		Var <-> Var		
2	Var -> distance<br>Var -> out<br>Var -> latency<br>Var -> cross	distance <-> distance<br>out <-> out<br>latency <-> latency<br>cross <-> cross<br>duration <-> duration<br>Var <-> Var	81.91	none
3	Var -> distance<br>Var -> out<br>Var -> latency<br>Var -> duration	distance <-> distance<br>out <-> out<br>latency <-> latency<br>cross <-> cross<br>duration <-> duration<br>Var <-> Var	87.44	none
4	Var -> distance<br>Var -> latency<br>Var -> cross<br>Var -> duration	distance <-> distance<br>out <-> out<br>latency <-> latency<br>cross <-> cross<br>duration <-> duration<br>Var <-> Var	62.13	none
5	Var -> out<br>Var -> latency<br>Var -> cross<br>Var -> duration	distance <-> distance<br>out <-> out<br>latency <-> latency<br>cross <-> cross<br>duration <-> duration<br>Var <-> Var	42.52	none
6	Var -> distance<br>Var -> out<br>Var -> cross<br>Var -> duration	distance <-> distance<br>out <-> out<br>latency <-> latency<br>cross <-> cross<br>duration <-> duration<br>Var <-> Var	104.99	none
7	Var1 -> distance<br>Var1 -> out<br>Var1 -> latency<br>Var2 -> cross<br>Var2 -> duration	distance <-> distance<br>out <-> out<br>latency <-> latency<br>cross <-> cross<br>duration <-> duration<br>Var1 <-> Var1<br>Var1 <-> Var2<br>Var2 <-> Var2	32.22	none
8	Var2 -> distance<br>Var1 -> out<br>Var1 -> latency<br>Var1 -> cross<br>Var2 -> duration	distance <-> distance<br>out <-> out<br>latency <-> latency<br>cross <-> cross<br>duration <-> duration<br>Var1 <-> Var1<br>Var1 <-> Var2<br>Var2 <-> Var2	33.27	none
9	Var2 -> distance<br>Var2 -> out<br>Var1 -> latency<br>Var1 -> cross<br>Var1 -> duration	distance <-> distance<br>out <-> out<br>latency <-> latency<br>cross <-> cross<br>duration <-> duration<br>Var1 <-> Var1<br>Var1 <-> Var2	33.42	none

		Var2 <-> Var2		
10	Var1 -> distance<br>Var1 -> out<br>Var2 -> latency<br>Var1 -> cross<br>Var2 -> duration	distance <-> distance<br>out <-> out<br>latency <-> latency<br>cross <-> cross<br>duration <-> duration<br>Var1 <-> Var1<br>Var1 <-> Var2<br>Var2 <-> Var2	33.31	none
11	Var1 -> distance<br>Var1 -> out<br>Var2 -> latency<br>Var2 -> cross<br>Var1 -> duration	distance <-> distance<br>out <-> out<br>latency <-> latency<br>cross <-> cross<br>duration <-> duration<br>Var1 <-> Var1<br>Var1 <-> Var2<br>Var2 <-> Var2	29.54	None
12	Var2 -> distance<br>Var1 -> out<br>Var2 -> latency<br>Var1 -> cross<br>Var1 -> duration	distance <-> distance<br>out <-> out<br>latency <-> latency<br>cross <-> cross<br>duration <-> duration<br>Var1 <-> Var1<br>Var1 <-> Var2<br>Var2 <-> Var2	29.02	none
13	Var1 -> distance<br>Var2 -> out<br>Var1 -> latency<br>Var1 -> cross<br>Var2 -> duration	distance <-> distance<br>out <-> out<br>latency <-> latency<br>cross <-> cross<br>duration <-> duration<br>Var1 <-> Var1<br>Var1 <-> Var2<br>Var2 <-> Var2	-	convergence
14	Var1 -> distance<br>Var2 -> out<br>Var1 -> latency<br>Var2 -> cross<br>Var1 -> duration	distance <-> distance<br>out <-> out<br>latency <-> latency<br>cross <-> cross<br>duration <-> duration<br>Var1 <-> Var1<br>Var1 <-> Var2<br>Var2 <-> Var2	33.03	none
15	Var1 -> distance<br>Var2 -> out<br>Var2 -> latency<br>Var1 -> cross<br>Var1 -> duration	distance <-> distance<br>out <-> out<br>latency <-> latency<br>cross <-> cross<br>duration <-> duration<br>Var1 <-> Var1<br>Var1 <-> Var2<br>Var2 <-> Var2	-	convergence
**16**	**Var2 -> distance**<br>**Var1 -> out**<br>**Var1 -> latency**<br>**Var2 -> cross**<br>**Var1 -> duration**	**distance <-> distance**<br>**out <-> out**<br>**latency <-> latency**<br>**cross <-> cross**<br>**duration <-> duration**	**27.56**	**none**

		**Var1 <-> Var1**<br>**Var1 <-> Var2**<br>**Var2 <-> Var2**		
17	Var1 -> distance<br>Var1 -> out<br>Var1 -> latency<br>Var2 -> cross<br>Var2 -> duration	distance <-> distance<br>out <-> out<br>latency <-> latency<br>cross <-> cross<br>duration <-> duration<br>Var1 <-> Var1<br>Var2 <-> Var2	-	singularity
18	Var2 -> distance<br>Var1 -> out<br>Var1 -> latency<br>Var1 -> cross<br>Var2 -> duration	distance <-> distance<br>out <-> out<br>latency <-> latency<br>cross <-> cross<br>duration <-> duration<br>Var1 <-> Var1<br>Var2 <-> Var2	-	singularity
19	Var2 -> distance<br>Var2 -> out<br>Var1 -> latency<br>Var1 -> cross<br>Var1 -> duration	distance <-> distance<br>out <-> out<br>latency <-> latency<br>cross <-> cross<br>duration <-> duration<br>Var1 <-> Var1<br>Var2 <-> Var2	-	singularity
20	Var1 -> distance<br>Var1 -> out<br>Var2 -> latency<br>Var1 -> cross<br>Var2 -> duration	distance <-> distance<br>out <-> out<br>latency <-> latency<br>cross <-> cross<br>duration <-> duration<br>Var1 <-> Var1<br>Var2 <-> Var2	-	singularity
21	Var1 -> distance<br>Var1 -> out<br>Var2 -> latency<br>Var2 -> cross<br>Var1 -> duration	distance <-> distance<br>out <-> out<br>latency <-> latency<br>cross <-> cross<br>duration <-> duration<br>Var1 <-> Var1<br>Var2 <-> Var2	-	singularity
22	Var2 -> distance<br>Var1 -> out<br>Var2 -> latency<br>Var1 -> cross<br>Var1 -> duration	distance <-> distance<br>out <-> out<br>latency <-> latency<br>cross <-> cross<br>duration <-> duration<br>Var1 <-> Var1<br>Var2 <-> Var2	-	singularity
23	Var1 -> distance<br>Var2 -> out<br>Var1 -> latency<br>Var1 -> cross<br>Var2 -> duration	distance <-> distance<br>out <-> out<br>latency <-> latency<br>cross <-> cross<br>duration <-> duration<br>Var1 <-> Var1<br>Var2 <-> Var2	-	singularity
24	Var1 -> distance<br>Var2 -> out	distance <-> distance<br>out <-> out	-	singularity

	Var1 -> latency<br>Var2 -> cross<br>Var1 -> duration	latency <-> latency<br>cross <-> cross<br>duration <-> duration<br>Var1 <-> Var1<br>Var2 <-> Var2		
25	Var1 -> distance<br>Var2 -> out<br>Var2 -> latency<br>Var1 -> cross<br>Var1 -> duration	distance <-> distance<br>out <-> out<br>latency <-> latency<br>cross <-> cross<br>duration <-> duration<br>Var1 <-> Var1<br>Var2 <-> Var2	-	singularity
26	Var2 -> distance<br>Var1 -> out<br>Var1 -> latency<br>Var2 -> cross<br>Var1 -> duration	distance <-> distance<br>out <-> out<br>latency <-> latency<br>cross <-> cross<br>duration <-> duration<br>Var1 <-> Var1<br>Var2 <-> Var2	-	singularity

Table S2. Estimates and test statistics for the Bayesian models looking at the effect of trial and life stage on the five behavioural variables measured in both tadpoles and metamorphs (full dataset). Results with a p-value below 0.05 are highlighted in bold.

Behaviour	Parameter	Posterior mean	Lower 95%CI	Upper 95%CI	p-value
Likelihood to exit the covered area	ID	6.031	0.0003	16.34	
	clutch	0.562	0.0002	2.093	
	(Intercept)	0.707	-7.789	9.419	0.856
	trial	5.362	1.893	9.435	**<0.001**
	life stage (tadpole)	0.894	-3.742	5.648	0.740
	Days to metamorphosis	-0.120	-0.302	0.035	0.124
	life stage*trial	-3.349	-7.195	0.179	**0.031**
Latency to exit the covered area (log transformation)	ID	0.047	0.0003	0.133	
	clutch	0.020	0.0002	0.073	
	(Intercept)	4.844	3.800	5.808	**<0.001**
	trial	-0.359	-0.578	-0.149	**<0.001**
	life stage (tadpole)	0.462	-0.121	1.069	0.128
	Days to metamorphosis	0.024	0.006	0.043	**0.008**
	life stage*trial	0.151	-0.099	0.407	0.247
Number of crossings between areas	ID	0.322	0.0004	0.876	
	clutch	0.065	0.0002	0.256	
	(Intercept)	1.965	-0.167	4.141	0.068
	trial	0.631	0.188	1.036	**0.003**
	life stage (tadpole)	-1.457	-2.744	-0.150	**0.026**
	Days to metamorphosis	-0.042	-0.084	-0.001	**0.045**
	life stage*trial	0.159	-0.328	0.676	0.525

Time spent in the uncovered area (constant transformation)	ID	3.124	0.0002	13.83	
	clutch	1.016	0.0002	5.03	
	(Intercept)	26.047	15.391	38.924	**<0.001**
	trial	3.229	0.436	6.216	**0.033**
	life stage (tadpole)	-12.550	-20.598	-4.464	**0.006**
	Days to metamorphosis	-0.299	-0.498	-0.089	**0.007**
	life stage*trial	0.166	-3.363	3.514	0.920
Distance travelled (constant transformation)	ID	0.893	0.0002	4.409	
	clutch	7.868	0.0001	24.34	
	(Intercept)	18.510	10.520	26.485	**<0.001**
	trial	-0.065	-1.916	1.740	0.934
	life stage (tadpole)	-4.052	-9.753	0.456	0.114
	Days to metamorphosis	-0.002	-0.157	0.131	0.998
	life stage*trial	2.332	0.203	4.509	**0.039**
